# The Novelty of Human Cancer/Testis Antigen Encoding Genes in Evolution

**DOI:** 10.1155/2013/105108

**Published:** 2013-04-18

**Authors:** Pavel Dobrynin, Ekaterina Matyunina, S. V. Malov, A. P. Kozlov

**Affiliations:** ^1^The Biomedical Center, Saint Petersburg 194044, Russia; ^2^Dobzhansky Center for Genome Bioinformatics, Saint Petersburg State University, Saint Petersburg 190004, Russia

## Abstract

In order to be inherited in progeny generations, novel genes should originate in germ cells. Here, we suggest that the testes may play a special “catalyst” role in the birth and evolution of new genes. Cancer/testis antigen encoding genes (CT genes) are predominantly expressed both in testes and in a variety of tumors. By the criteria of evolutionary novelty, the CT genes are, indeed, novel genes. We performed homology searches for sequences similar to human CT in various animals and established that most of the CT genes are either found in humans only or are relatively recent in their origin. A majority of all human CT genes originated during or after the origin of Eutheria. These results suggest relatively recent origin of human CT genes and align with the hypothesis of the special role of the testes in the evolution of the gene families.

## 1. Introduction

In order to be inherited in progeny generations, novel genes should originate in germ cells. Available data suggest that the generation of novel genes in germ cells is ongoing process, for example, the promiscuity of gene expression in spermatogenic cells [1, 2]. Novel genes may originate through different mechanisms (retrogenes, segmental duplicates, chimeric, and *de novo* emerged genes), but all of them are uniformly expressed in the testis ([3–8]; reviewed in [9]). These observations led us to suggest that testes may play a “tissue catalyst” role in the birth and evolution of new genes [9]. Previously, we proposed the expression of evolutionarily novel genes in tumors [10].

Cancer/testis or cancer/germline antigen genes are a class of genes with predominant expression in testis and in a variety of tumors, with a significant exclusion of some CT antigens also expressed in the brain. Here we set forth to test the hypothesis that cancer/testis antigen genes should be composed of evolutionarily new or young gene family. We performed homology searches for sequences similar to human CT in various animals. Additionally, as an extensive traffic of novel genes has been described for mammalian X chromosome [3, 6, 11], we also performed this analysis separately for genes located on this chromosome only.

## 2. Methods

The list of CT antigens gene was retrieved from CT Database (http://www.cta.lncc.br) and included 265 genes. Among them, there are 105 CT antigens that are encoded by the X chromosome (CT-X genes) and 105 that are located on various autosomes (autosome CT genes, or non-X CT genes). Eight CT antigen encoding genes are located on the Y chromosome.

To assess the evolutionary novelty of the studied group of CT genes by searching orthologues for each of CT genes, the HomoloGene.release 66 (http://www.ncbi.nlm.nih.gov/homologene/) tool from NCBI website was used. HomoloGene is a database of both curated and computed gene orthologs and orthologues and now covers 21 organisms. Curated orthologs include gene pairs from the Mouse Genome Database (MGD) at the Jackson Laboratory, the Zebrafish Information (ZFIN) database at the University of Oregon, and from published reports. Computed orthologs and orthologues, which are considered putative, are identified from BLAST nucleotide sequence comparisons between all UniGene clusters for each pair of organisms [12]. As an input, the program uses gene name and/or taxon name, and the output is clusters of orthologues. For this study, the search was performed in several completely sequenced eukaryotic genomes, including *H. sapiens, P. troglodytes, M. mulatta, C. lupus, B. taurus, M. musculus, R. norvegicus, G. gallus, D. rerio, D. melanogaster, A. gambiae, C. elegans, S. cerevisiae, K. lactis, A. gossypii, S. pombe, M. oryzae, N. crassa, A. thaliana, O. sativa,* and *P. falciparum*. 

According to the origin of their orthologues in different taxa of human phylogeny, the CT genes and all human genes were distributed into 11 groups. The differences in distribution of CT genes and all human genes were assessed using the chi square test [13]. Sheffe's S method of multiple estimation ([14, 15]; for counts see also [16]) was applied to define the difference and to show stochastically that the origin of human CT genes is substantially more recent than that for all human genes.

## 3. Results

The results obtained using HomoloGene tool applied to human CT genes are presented in Table 1. The full list of studied CT genes is present in Supplementary material (see Supplementary Material available online at http://dx.doi.org/10.1155/2013/105108. HomoloGene assigned each gene to a certain homology group which includes orthologues from different taxa within human lineage. Of 265 genes represented in CT Database, 47 did not match any homology group, probably because of the differences in the gene names making matches with HomoloGene database difficult. Human CT genes orthologues are widely distributed throughout the human lineage. For example, for one CT-X gene (*FAM133A*), the orthologues were found in all Eukaryota, and for two CT-X genes (*MAGEC1* and *SPANXN4*), the orthologues were first found in Bilateria, and for three CT-X genes (*ARX*, *IL13RA,* and *FAM46D*), the time of origin was placed in Euteleostomi. There were substantially larger numbers of CT-X genes with orthologues emerging in Eutheria, Catarrhini, and Homininae and of CT-X genes that were found exclusively in humans. Interestingly, there was a Eutheria-specific subfamily TSPY1 composed of 8 CT genes and located on chromosome Y. 

**Figure 1:**
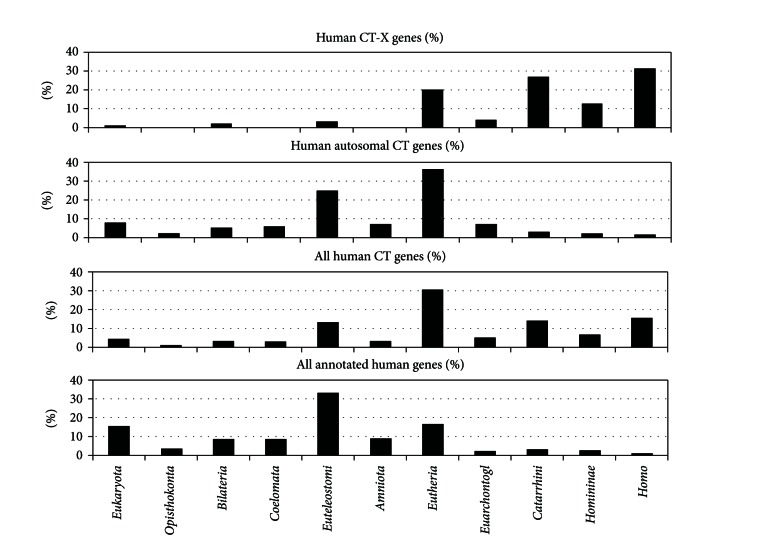
The proportions of CT-X genes, autosomal CT genes, all human CT genes, and all annotated human genes with orthologues originated in different taxa of *H. sapiens* lineage.

Similarly searches for the orthologues were performed for all CT-X genes, all autosomal CT genes, all human CT genes, and all annotated protein coding genes in human genome (assembly GRCh37) (Table 2 and Figure 1). 

The results show that the proportion of autosomal CT genes that has orthologues originated in Euteleostomi and in Eutheria (24.8% and 36.2%, accordingly) is greater than that on chromosome X. Only a few of autosomal CT genes are exclusive for humans. We found that CT gene *POTEB* (prostate, ovary, testis-expressed protein on chromosome 15, Ensembl: ENSG00000233917) has a poorly characterized homologue (LOC100287399, Ensembl: ENSG00000230031) that is according to HomoloGene criteria is exclusive to *H. sapiens*. This newly described homolog (LOC100287399, Ensembl: ENSG00000230031) has not been previously annotated as a gene of CT family.

 Among all annotated human protein coding genes, the proportion of genes specific to humans only is very small (0.85%). The list of these human-specific genes includes 163 entries, 33 of which are CT-X genes.

For CT-X genes, the distribution was different: 31.4% of CT-X genes (five *CT45A* genes, twelve *CT47A* genes, fifteen *GAGE* genes, and four *XAGE* genes) are present in humans only, while 39.1% of CT-X genes have orthologues that emerged in *Catarrhini* or *Homininae*. This means that the majority (70.5%) of CT-X genes present in human genome are either novel or relatively recent. At the same time, distribution of all genes located on X chromosome is similar to that for all human genes (see Supplementary Table IV).

The distribution of all human CT genes shows that 30.73% of CT genes have orthologues that originated in *Eutheria*. This proportion is larger than the proportion of all human genes with pan-*Eutherian* orthologues (16.41%). Importantly, 36.7% of all human CT genes originated in *Catarrhini*, *Homininae,* or humans. Thus, the majority of human CT genes (72.48%) originated during or after the emergence of Eutheria. On the other side, the majority of annotated human genes (75.95%) were older than *Eutheria*.

**Figure 2:**
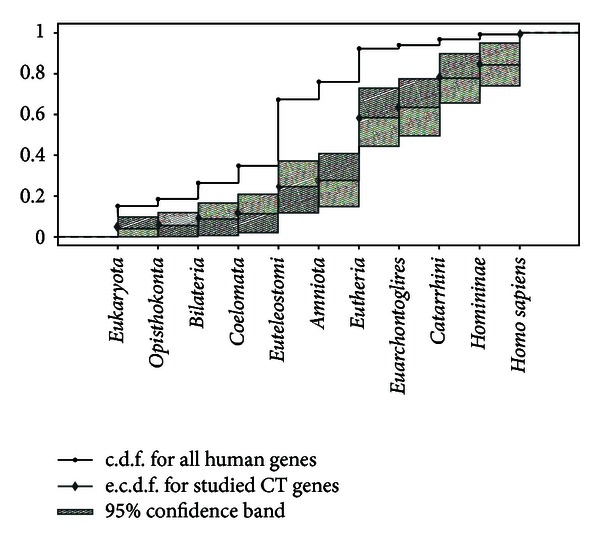
Cumulative distribution function for all human genes and empirical distribution function for all CT human genes, in accordance with the origin of their orthologues in different taxa, with 95% confidence bands. c.d.f.—cumulative distribution function. e.c.d.f.—empirical cumulative distribution function.

A significance of the difference between distribution of all human genes and all human CT genes according to the origin of their orthologues in different taxa was confirmed bychi square test (*P* value less than 10^−6^). Moreover, 95% confidence region for the cumulative distribution function of CT human genes displays that CT genes are stochastically younger as compared to all human genes. In other words, the probability that a gene randomly chosen from all human genes is younger than some fixed time *T* is less than the probability that a randomly chosen CT gene is younger than *T*. Therefore, there is a significant bias in time of origin for human CT genes as compared to all human genes. If human CT genes would be obtained as a sample from some probabilistic distribution, the probability that CT human genes originated not earlier than *Catarrhini* or *Eutheria *would be significantly higher than the respective probability for census of all human genes (Figure 2). This statistical trial confirms that the origin of human CT genes is relatively recent.

## 4. Conclusion

Cancer/testis antigen genes (CTA or CT genes) encode a subgroup of tumor antigens expressed predominantly in testis and various tumors. CT antigens may be also expressed in placenta and in female germ cells [17–20]. In addition, some CT antigens are expressed in the brain [21].

Experimentally, human CT genes were discovered by a variety of immunological screening methods [22], serological identification of antigens by recombinant expression cloning (SEREX) [23], expression database analysis [24, 25], massively parallel signature sequencing [26], and other approaches. The fact that many CT antigens have been identified using SEREX suggests that they are highly antigenic [23, 27].

The first CT gene discovered was *MAGEA1* that encodes for an antigen of human melanoma [22]. This gene belongs to a family of 12 closely related genes clustered at Xq28. A second cluster of *MAGE* genes, *MAGEB*, was discovered at Xp21.3, and the third, encoding *MAGEC* genes, is located at Xq26-27. The expression of *MAGEA-MAGEC* genes (*MAGE-I* subfamily) is restricted to testis and cancer, whereas more distantly related clusters *MAGED-MAGEL* (subfamily *MAGE-II*) are expressed in many normal tissues. *MAGE-I* genes are of relatively recent origin, and *MAGE-II* genes are relatively more ancient. For example, *MAGE-D* genes are conserved between man and mouse. One of these genes corresponds to the founder member of the family, and the other *MAGE* genes are retrogenes derived from the common ancestral gene [19, 28, 29]. 

To date, CTD atabase (http://www.cta.lncc.br/) includes 265 CT genes. More than half of them are located on X-chromosome (CT-X genes) [21]. The analysis of the DNA sequence of the human X chromosome predicts that approximately 10% of the genes on the X chromosome are of the CT antigen type [30]. Non-X CT genes are distributed throughout the genome and are represented mainly by single-copy genes [19, 27, 31]. 

In normal testis, CT-X genes are expressed in proliferating germ cells (spermatogonia). Non-X CT genes are expressed during later stages of germ-cell differentiation, that is, spermatocytes [19]. Among human tumors, CT antigens are expressed in melanoma, bladder cancer, lung cancer, breast cancer, prostate cancer, sarcoma, ovarian cancer, hepatocellular carcinoma, hematologic malignancies, and so forth [21, 27, 31, 32]. Genome-wide analysis of 153 cancer/testis genes expression has led to their classification into testis-restricted (*N* = 39), testis/brain-restricted (*N* = 14) and testis-selective (*N* = 85) groups of genes, the latter group showing some expression in nongermline tissues. The majority of testis-restricted genes belong to CT-X group (35 of total 39 testis-restricted groups), while non-X CT genes are expressed in a less restrictive way [21].

Multiple CT antigens are often coexpressed in tumors suggesting that this expression program is coordinated for entire family [19, 23, 33]. CT gene expression is controlled by epigenetic mechanisms which include DNA methylation and histone posttranslational modifications [31]. Other mechanisms of CT gene regulation include sequence-specific transcription factors and signal transduction pathways such as activated tyrosine kinases [34]. 

The functions of CT-X genes are largely unknown. On the contrary, more is known about functions of non-X CT genes which are associated with meiosis, gametogenesis, and fertilization. Non-X CTs are also more conserved during evolution [21, 27, 31, 32].

CT-X genes tend to form recently expanded gene families, many with nearly identical gene copies [17–20, 26, 32, 35]. 

The prevalence of large, highly homologous inverted repeats (IRs) containing testes genes on the X- and Y-chromosomes was described in humans and great apes [36, 37]. CT-X gene families are also located in direct or inverted repeats [20].

The study of clusters of homologous genes originated by gene duplication roughly after the divergence of the human and rodent lineages discovered several families of CT genes among recent duplicates [38].

In the other paper, the authors also studied recent duplications in the human genome and found that CT genes were represented in this gene set, including the family of *PRAME* (preferentially expressed antigen of melanoma) genes located on chromosome 1 and expressed in the testis and in a large number of tumors [39]. Duplicated *PRAME* genes are hominid specific, having arisen in human genome since the divergence from chimps. *PRAME* gene family also expanded in other *Eutheria*. Chimp and mouse have orthologous *PRAME* gene clusters on their chromosomes 1 and 4, respectively [39, 40].

Rapid evolution of cancer/testis genes has been demonstrated on the X chromosome. In particular, the comparison of human: chimp orthologues of these genes has shown that they diverge faster and undergo stronger positive selection than those on the autosomes or than control genes on either X chromosome or autosomes [41]. 

*SPANX-A/D* gene subfamily of cancer/testis-specific antigens evolved in the common ancestor of the hominoid lineage after its separation from orangutan. Southern blot and database analyses have detected *SPANX* sequences only in primates [17]. The coding sequences of the *SPANX* genes evolved rapidly, faster than their introns and the 5′ untranslated regions, with accelerated rates of substitutions in both synonymous and nonsynonymous codon positions. The mechanism of *SPANX* genes expansion was segmental DNA duplications, with evidence of positive selection. *SPANX-N* is the ancestral form, from which the *SPANX-A/D* subfamily evolved in the common ancestor of hominoids approximately 7 MYA [35, 42]. *SPANX* genes are expressed in cancer cells and highly metastatic cell lines from melanomas, bladder carcinomas, and myelomas [35].

The *GAGE* cancer/testis antigen gene family contains at least 16 genes which are encoded by an equal number of tandem repeats. All *GAGE* genes are located at Xp11.23. *GAGE* genes are highly identical and evolved under positive selection that supports their recent origin [43, 44].

The *XAGE* family of cancer/testis antigen genes belongs to superfamily of *GAGE-*like CT genes. It is located on chromosome Xp11.21-Xp11.22. Three *XAGE* genes are described, as well as several splice variants of *XAGE-1* [45, 46].

*CT45* gene family was discovered by massively parallel signature sequencing. It includes six highly similar (>98%) genes that are cluctered in tandem on chromosome Xq26.3. CT45 antigen is expressed in Hodgkin's lymphoma and in other human tumors [26, 47–49]. 

*CT47* cancer/testis gene family is located on chromosome Xq24. Among normal tissues, it is expressed in the testis and (weakly) in placenta and brain. In tumors, its expression was found in lung cancer and esophageal cancer. The *CT47* family member is characterized by high (>98%) sequence homology. Chimp is the only other species in which a gene homologous to *CT47* was found by other authors [20].

Our work is the first systematic study of the evolutionary novelty of the whole class of CT genes. To assess the evolutionary novelty of CT genes, we applied the HomoloGene tool of NCBI. To construct the clusters of orthologues, the HomoloGene program uses information from blastp, phylogenetic analyses, and syntheny information when it is possible. Cutoffs on bits per position and Ks values are set to prevent unlikely “orthologs” from being grouped together. These cutoffs are calculated based on the respective score distribution for the given groups of organisms [12].

We searched for orthologues of each of CT genes among annotated genes in several completely sequenced eukaryotic genomes and built distributions of all CT-X genes, all autosomal CT genes, all human CT genes, and all annotated protein coding genes from human genome according to the origin of their orthologues in 11 taxa of human lineage. 

 We have shown that 31.4% of CT-X genes are exclusive for humans and 39.1% of CT-X genes have orthologues originated in *Catarrhini* or* Homininae*. Thereby, the majority of human CT-X genes (70.5%) are novel or recent in its origin. Our data are in good correspondence with evidence obtained by other groups on rapid expansion of certain CT-X gene families and high homology of their members which suggest their recent origin. 

Altogether 36.7% of all human CT genes originated in *Catarrhini, Homininae,* and humans. We have also found that 30.73% of all human CT genes originated in *Eutheria*. These CT genes acquired functions in *Eutheria*. This indicates the importance of processes in which tumors and CT antigens were involved during the evolution of* Eutheria*. CT genes originated in *Eutheria* are located mostly on autosomes. CT genes originated in *Catarrhini, Homininae,* and humans are located predominantly on X chromosome. This difference is probably related to evolution of mammalian X chromosome since the origin of *Eutheria* [50], especially to the acquisition of its special role in the origin of novel genes [9].

Thus, the majority of CT-X genes are either novel or young for humans, and the majority of all human CT genes (72.48%) originated during or after the origin of *Eutheria*. These results suggest that the whole class of human CT genes is relatively evolutionarily new.

In its turn, this conclusion confirms our prediction about expression of evolutionary recent and novel genes in tumors [10]. The expression of cancer/testis genes in tumors is then a natural phenomenon, not aberrant process as suggested by many authors (e.g., [19, 27, 32, 34, 40]). 

## Supplementary Material

Supplement materials can be found in Excel file. Data in this file divided into three sheets. The first contains information about gene families, gene common names, chromosomes positions and novelty of all Cancer testis genes that we were able to find in ENSEMBL database. Second is a list of CT gene names and their ENSEMBL id's. And third is a list of all CT genes that were presented in CT database but we were failed to find them in ENSEMBL database.

## Figures and Tables

**Table 1:** Distribution of all human CT genes according to the origin of their orthologues in different taxa of human lineage.

Taxa	Chromosome names																							
	1	2	3	4	5	6	7	8	9	10	11	12	13	14	15	16	17	18	19	20	21	22	X	Y
Eukaryote	1	1					1			2			1							1	1		1	
Opisthokonta	1									1														
Bilateria		1				1		1									2						2	
Coelomata						2		2	1												1			
Euteleostomi	4	4	2	1	1	2		2		3	1	2		1	1	1			1				3	
Amniota	3														2		2							
Eutheria	2		3	3	1	2		4	3		3	2			1	1	3	1	6	2		1	21	8
Euarchontoglires		1												1		1		1	1	1		1	4	
Catarrhini																		1		1	1		28	
Homininae								1										1					13	
*Homo sapiens*															1								33	

Total	11	7	5	4	2	7	1	10	4	6	4	4	1	2	5	3	7	4	8	5	3	2	105	8

**Table 2:** CT-X genes, autosomal CT genes, all human CT genes, and all annotated human genes with orthologues originated in different taxa of *H. sapiens *lineage.

Taxa	CT-X genes		Autosome CT genes		All CT genes		All human genes	
Eukaryota	1,0%	1	7,6%	8	4,13%	9	15,19%	2900
Opisthokonta			1,9%	2	0,92%	2	3,21%	613
Bilateria	1,9%	2	4,8%	5	3,21%	7	8,12%	1549
Coelomata			5,7%	6	2,75%	6	8,27%	1579
Euteleostomi	2,9%	3	24,8%	26	13,30%	29	32,77%	6256
Amniota			6,7%	7	3,21%	7	8,39%	1601
Eutheria	20,0%	21	36,2%	38	30,73%	67	16,41%	3132
Euarchontoglires	3,8%	4	6,7%	7	5,05%	11	1,75%	334
Catarrhini	26,7%	28	2,9%	3	14,22%	31	2,66%	507
Homininae	12,4%	13	1,9%	2	6,88%	15	2,38%	454
Homo sapiens	31,4%	33	1,0%	1	15,60%	34	0,85%	163

Total	100,0%	105	100,0%	105	100,00%	218	100,00%	19088

## References

[1] Schmidt EE (1996). Transcriptional promiscuity in testes. *Current Biology*.

[2] Kleene KC (2005). Sexual selection, genetic conflict, selfish genes, and the atypical patterns of gene expression in spermatogenic cells. *Developmental Biology*.

[3] Betrán E, Thornton K, Long M (2002). Retroposed new genes out of the X in Drosophila. *Genome Research*.

[4] Paulding CA, Ruvolo M, Haber DA (2003). The *Tre2* (USP6) oncogene is a hominoid-specific gene. *Proceedings of the National Academy of Sciences of the United States of America*.

[5] She X, Cheng Z, Zöllner S, Church DM, Eichler EE (2008). Mouse segmental duplication and copy number variation. *Nature Genetics*.

[6] Levine MT, Jones CD, Kern AD, Lindfors HA, Begun DJ (2006). Novel genes derived from noncoding DNA in Drosophila melanogaster are frequently X-linked and exhibit testis-biased expression. *Proceedings of the National Academy of Sciences of the United States of America*.

[7] Heinen TJAJ, Staubach F, Häming D, Tautz D (2009). Emergence of a new gene from an intergenic region. *Current Biology*.

[8] Kaessmann H, Vinckenbosch N, Long M (2009). RNA-based gene duplication: mechanistic and evolutionary insights. *Nature Reviews Genetics*.

[9] Kaessmann H (2010). Origins, evolution, and phenotypic impact of new genes. *Genome Research*.

[10] Kozlov AP (2010). The possible evolutionary role of tumors in the origin of new cell types. *Medical Hypotheses*.

[11] Emerson JJ, Kaessmann H, Betrán E, Long M (2004). Extensive gene traffic on the mammalian X chromosome. *Science*.

[12] Sayers EW, Barrett T, Benson DA (2012). Database resources of the National Center for Biotechnology Information. *Nucleic Acids Research*.

[13] R Development Core Team (2011). *R: A Language and Environment for Statistical Computing*.

[14] Sheffe H (1959). *The Analysis of Variance*.

[15] Sheffe H (1970). Multiple testing versus multiple estimation. Improper confidence sets. Estimation of directions and ratios. *The Annals of Mathematical Statistics*.

[16] Goodman LA (1964). Simultaneous confidence intervals for contrasts among multinomial populations. *The Annals of Mathematical Statistics*.

[17] Zendman AJW, Zschocke J, Van Kraats AA (2003). The human SPANX multigene family: genomic organization, alignment and expression in male germ cells and tumor cell lines. *Gene*.

[18] Zendman AJW, Ruiter DJ, Van Muijen GNP (2003). Cancer/testis-associated genes: identification, expression profile, and putative function. *Journal of Cellular Physiology*.

[19] Simpson AJG, Caballero OL, Jungbluth A, Chen YT, Old LJ (2005). Cancer/testis antigens, gametogenesis and cancer. *Nature Reviews Cancer*.

[20] Chen YT, Iseli C, Yenditti CA, Old LJ, Simpson AJG, Jongeneel CV (2006). Identification of a new cancer/testis gene family, CT47, among expressed multicopy genes on the human X chromosome. *Genes Chromosomes and Cancer*.

[21] Hofmann O, Caballero OL, Stevenson BJ (2008). Genome-wide analysis of cancer/testis gene expression. *Proceedings of the National Academy of Sciences of the United States of America*.

[22] Van Der Bruggen P, Traversari C, Chomez P (1991). A gene encoding an antigen recognized by cytolytic T lymphocytes on a human melanoma. *Science*.

[23] Sahin U, Tereci O, Schmitt H (1995). Human neoplasms elicit multiple specific immune responses in the autologous host. *Proceedings of the National Academy of Sciences of the United States of America*.

[24] Scanlan MJ, Gordon CM, Williamson B (2002). Identification of cancer/testis genes by database mining and mRNA expression analysis. *International Journal of Cancer*.

[25] Scanlan MJ, Simpson AJ, Old LJ (2004). The cancer/testis genes: review, standardization, and commentary. *Cancer Immunity*.

[26] Chen YT, Scanlan MJ, Venditti CA (2005). Identification of cancer/testis-antigen genes by massively parallel signature sequencing. *Proceedings of the National Academy of Sciences of the United States of America*.

[27] Cheng YH, Wong EWP, Cheng CY (2011). Cancer/testis (CT) antigens, carcinogenesis and spermatogenesis. *Spermatogenesis*.

[28] Chomez P, De Backer O, Bertrand M, De Plaen E, Boon T, Lucas S (2001). An overview of the MAGE gene family with the identification of all human members of the family. *Cancer Research*.

[29] Katsura Y, Satta Y (2011). Evolutionary history of the cancer immunity antigen MAGE gene family. *PLoS ONE*.

[30] Ross MT, Grafham DV, Coffey AJ (2005). The DNA sequence of the human X chromosome. *Nature*.

[31] Fratta E, Coral S, Covre A (2011). The biology of cancer testis antigens: putative function, regulation and therapeutic potential. *Molecular Oncology*.

[32] Caballero OL, Chen YT (2009). Cancer/testis (CT) antigens: potential targets for immunotherapy. *Cancer Science*.

[33] Sahin U, Tureci O, Chen YT (1998). Expression of multiple cancer/testis antigens in breast cancer and melanoma: basis for polyvalent CT vaccine strategies. *International Journal of Cancer*.

[34] Akers SN, Odunsi K, Karpf AR (2010). Regulation of cancer germline antigen gene expression: implications for cancer immunotherapy. *Future Oncology*.

[35] Kouprina N, Mullokandov M, Rogozin IB (2004). The SPANX gene family of cancer/testis-specific antigens: rapid evolution and amplification in African great apes and hominids. *Proceedings of the National Academy of Sciences of the United States of America*.

[36] Skaletsky H, Kuroda-Kawaguchl T, Minx PJ (2003). The male-specific region of the human Y chromosome is a mosaic of discrete sequence classes. *Nature*.

[37] Warburton PE, Giordano J, Cheung F, Gelfand Y, Benson G (2004). Inverted repeat structure of the human genome: the X-chromosome contains a preponderance of large, highly homologous inverted repeated that contain testes genes. *Genome Research A*.

[38] IHGSC (2004). International human genome sequencing consortium. *Nature*.

[39] Birtle Z, Goodstadt L, Ponting C (2005). Duplication and positive selection among hominin-specific PRAME genes. *BMC Genomics*.

[40] Chang TC, Yang Y, Yasue H, Bharti AK, Retzel EF, Liu WS (2011). The expansion of the PRAME gene family in Eutheria. *PLoS ONE*.

[41] Stevenson BJ, Iseli C, Panji S (2007). Rapid evolution of cancer/testis genes on the X chromosome. *BMC Genomics*.

[42] Kouprina N, Noskov VN, Pavlicek A (2007). Evolutionary diversification of SPANX-N sperm protein gene structure and expression. *PLoS ONE*.

[43] Liu Y, Zhu Q, Zhu N (2008). Recent duplication and positive selection of the GAGE gene family. *Genetica*.

[44] Gjerstorff MF, Ditzel HJ (2008). An overview of the GAGE cancer/testis antigen family with the inclusion of newly identified members. *Tissue Antigens*.

[45] Zendman AJW, Van Kraats AA, Weidle UH, Ruiter DJ, Van Muijen GNP (2002). The XAGE family of cancer/testis-associated genes: alignment and expression profile in normal tissues, melanoma lesions and Ewing’s sarcoma. *International Journal of Cancer*.

[46] Sato S, Noguchi Y, Ohara N (2007). Identification of XAGE-1 isoforms: predominant expression of XAGE-1b in testis and tumors. *Cancer Immunity*.

[47] Chen YT, Hsu M, Lee P (2009). Cancer/testis antigen CT45: analysis of mRNA and protein expression in human cancer. *International Journal of Cancer*.

[48] Chen YT, Chadburn A, Lee P (2010). Expression of cancer testis antigen CT45 in classical Hodgkin lymphoma and other B-cell lymphomas. *Proceedings of the National Academy of Sciences of the United States of America*.

[49] Heidebrecht HJ, Claviez A, Kruse ML (2006). Characterization and expression of CT45 in Hodgkin's lymphoma. *Clinical Cancer Research*.

[50] Lahn BT, Page DC (1999). Four evolutionary strata on the human X chromosome. *Science*.

